# The effect of the physical exercise intervention on negative emotions in children and adolescents with neurodevelopmental disorders: a systematic review and meta-analysis

**DOI:** 10.3389/fpsyt.2025.1670044

**Published:** 2025-10-13

**Authors:** Anning Wang, Yingying Peng, Zhibo Cui, Qiubo Zhang, Tong Wang

**Affiliations:** ^1^ College of Sports Science, Qufu Normal University, Jining, Shandong, China; ^2^ College of Basic Teaching, Chengdu Neusoft University, Chengdu, Sichuan, China; ^3^ School of Sports Training, Chengdu Sport University, Chengdu, Sichuan, China; ^4^ Primary School Attached to Chengdu Normal College, Chengdu, Sichuan, China

**Keywords:** physical exercise, adolescents and children, negative emotions, neurodevelopmental disorders, systematic review and meta-analysis

## Abstract

**Objective:**

This systematic review and meta-analysis aimed to synthesize existing evidence on the association between physical exercise (PE) interventions and negative emotions (NEs), including anxiety and depressive symptoms, among adolescents and children with neurodevelopmental disorders (NDDs), while carefully considering sample size limitations and evidence heterogeneity.

**Methods:**

Relevant studies were obtained from PubMed, EBSCO, Cochrane, Web of Science, Embase, and PsyINFO databases up to 27 June, 2025. Key search terms included NEs, PE, adolescents and children, and NDDs. Meta-analysis was performed utilizing Review Manager 5.3, applying a random-effects model to estimate the standardized mean difference (SMD) along with 95% confidence intervals (CI). Subgroup analyses were conducted based on the type of negative emotion, intervention modality, duration, timing, and frequency.

**Results:**

PE interventions were associated with a statistically significant overall reduction in NEs in adolescents and children with NDDs (SMD = -0.60, 95% CI: -1.02 to -0.18, p < 0.01, Z = 2.80, I² = 79%). The effect was most evident for anxiety (SMD = -0.56, 95% CI: -1.11 to -0.00, p = 0.05, I² = 83%), suggesting a potential reduction in anxiety symptoms that approaches statistical significance. However, the findings for depressive symptoms were inconclusive (SMD = -0.82, 95% CI: -2.00 to -0.36, p = 0.17, I² = 87%), likely due to heterogeneity in interventions, small sample sizes, and variability in measurement tools. Subgroup analysis suggested that engaging in mixed forms of PE for a minimum of 60 minutes per session, once weekly, over a 12-week period, was especially effective in improving emotional well-being in this population.

**Conclusion:**

This study highlights the potential of PE interventions to alleviate negative emotions—especially anxiety—among adolescents and children with NDDs, but emphasizes that evidence for depressive symptoms remains inconclusive. The findings should be interpreted with caution due to limited study numbers, predominance of ADHD-focused samples, heterogeneity across studies, and reliance on subjective assessment tools. Future high-quality, adequately powered randomized controlled trials are warranted to confirm these effects and clarify optimal intervention parameters.

**Systematic review registration:**

https://www.crd.york.ac.uk/PROSPERO/recorddashboard, identifier CRD420251081387

## 1 Introduction

Neurodevelopmental disorders (NDDs) represent a broad spectrum of conditions typically emerging in early childhood, characterized by substantial limitations in autonomy, social integration, and academic or occupational functioning (1). Autism spectrum disorder (ASD), attention-deficit/hyperactivity disorder (ADHD), intellectual disability (ID), and specific learning disorders are included among these disorders, but are not limited to them. While prevalence rates differ by region and study, a consistent trend over the past two decades shows a marked increase in these conditions, emphasizing the need for greater awareness, early detection, and the development of innovative therapeutic approaches (2).

Negative emotions (NEs) reflect psychological distress and are often categorized by intensity, commonly including symptoms such as anger, sadness, depression, and anxiety (3). Among these, depression and anxiety are often utilized as key indicators of emotional distress (4). Depression is often chronic and recurrent (5), linked with significant psychological burden, functional impairment, and deteriorating physical health (6). It is the most prevalent component of negative influence and a leading contributor to global disease burden among individuals under 25 (7). It is estimated that approximately 20% of individuals will experience depression during adolescence or early adulthood, with onset typically occurring at some point in their lives (8, 9). Anxiety, on the other hand, is one of the most widespread mental health concerns in youth, adversely impacting academic achievement, social relationships, and adaptive functioning, while also increasing long-term mental health risks (10, 11). Research by Barkley et al. (12) and Meinzer et al. (13) demonstrated elevated levels of stress, depression, and anxiety in ADHD children. Further evidence from cross-sectional, retrospective, and longitudinal studies specifies that ADHD children are at elevated risk for comorbid psychiatric conditions throughout development, such as mood and anxiety disorders, as well as substance use disorders (14). Given this, depression and anxiety are vital to the construct of NEs and serve as the focal outcomes of this study.

Physical exercise (PE) includes a variety of activities such as aerobic training, resistance exercises, and combinations of physical and mental challenges (15). A growing body of literature supports the role of PE in enhancing mental well-being and easing emotional distress (16, 17). Meta-analyses have highlighted PE as a viable standalone or adjunctive intervention for improving internalizing and externalizing symptoms, cognitive functioning, and psychological well-being in adolescents and children with NDDs (18). In particular, PE has demonstrated beneficial effects on anxiety, depression, and executive functioning in young individuals with neurodevelopmental conditions (19, 20). Mechanistically, regular PE may induce neurobiological changes by enhancing dopamine, serotonin, and norepinephrine levels, contributing to improved emotional regulation (21). Previous interventions targeting typically developing adolescents and adults have shown that exercise can alleviate anxiety symptoms related to agoraphobia, post-traumatic stress disorder, separation anxiety, panic disorder, and generalized anxiety disorder (22–25). Notably, the anxiolytic effects of exercise may be comparable to those achieved through cognitive behavioral therapy or pharmacological treatment (24). Moreover, several studies have identified reductions in depressive and stress-related symptoms following structured exercise programs (26, 27). One proposed mechanism involves the modulation of dopamine (DA) dysregulation, a key factor in ADHD pathophysiology that also contributes to depression and stress (28–30). Thus, PE may offer a promising intervention for managing depression and anxiety in children affected by NDDs.

Compared with previous studies, this systematic review and meta-analysis distinctively highlights the nuanced effects of PE interventions on specific types of NEs (depression and anxiety) in adolescents and children with NDDs. Notably, the study incorporates a comprehensive subgroup analysis examining multiple intervention characteristics, including types of PE intervention, intervention duration, time, and frequency. This innovative and detailed analysis provides deeper insights into how various exercise parameters distinctly influence emotional outcomes, offering clear guidance for tailored exercise prescriptions and clinical practice, thereby significantly enriching existing literature and practical intervention strategies. As far as we know, currently there are no studies that have evaluated the impact of PE intervention measures for adolescents and children with NDDs on NEs. Accordingly, this review seeks to consolidate existing research findings on the impact of PE interventions in alleviating NEs among adolescents and children with NDDs.

## 2 Methods

This review was carried out in accordance with the PRISMA guidelines (31) and the “Cochrane Handbook for Systematic Reviews of Interventions” (32). Additionally, the review protocol was registered with PROSPERO (Registration No. CRD420251081387).

### 2.1 Search strategy

A systematic search was performed across six databases, Web of Science (WoS), EBSCO, PubMed, Cochrane, Embase, and PsycINFO, on June 27, 2025, to identify relevant studies. The search terms used are summarized in **Table 1**. A Boolean logic search strategy was employed, wherein the keywords were connected using “AND”, while each individual search term was linked using “OR”. The primary keywords included: “adolescents and children”, “negative emotions”, “neurodevelopmental disorders”, and “PE”. The detailed search formulation was as follows:

**Table 1:** Summary of search terms.

Category	Connective Words	Included search terms
Physical exercise		“aerobic training” OR “aerobic exercise” OR “athletic sports” OR “fitness game” OR “muscle-strengthening exercise” OR “resistance exercise” OR “physical education” OR “PE” OR “sports game” OR sport OR strength OR motor
	AND	
Neurodevelopmental disorder		“attention-deficit/hyperactivity disorder” OR ADD OR ADHD OR ASD OR “autism asperger” OR “autism spectrum disorder” OR “communication disorder” OR “developmental coordination disorder” OR “developmental disorder” OR “down syndrome” OR “intellectual disability” OR “learning disability” OR “motor disorder” OR “neurodevelopmental disorder” OR “pervasive developmental disorder not otherwise specified” OR “specific learning disorder”
	AND	
Negative emotion		“depress” OR “depressive” OR “depression” OR “anxiety” OR “anxious” OR “mental disease” OR “psychological ill-being” OR “NEs”
	AND	
Children and adolescents		(“junior high school students” OR “senior high school students” OR adolescent OR teenager)

(“aerobic training” OR “aerobic exercise” OR “athletic sports” OR “fitness game” OR “muscle-strengthening exercise” OR “resistance exercise” OR “physical education” OR “PE” OR “sports game” OR sport OR strength OR motor)

AND (“attention-deficit/hyperactivity disorder” OR ADD OR ADHD OR ASD OR “autism asperger” OR “autism spectrum disorder” OR “communication disorder” OR “developmental coordination disorder” OR “developmental disorder” OR “down syndrome” OR “intellectual disability” OR “learning disability” OR “motor disorder” OR “neurodevelopmental disorder” OR “pervasive developmental disorder not otherwise specified” OR “specific learning disorder”)

AND

(“depress” OR “depressive” OR “depression” OR “anxiety” OR “anxious” OR “mental disease” OR “psychological ill-being” OR “NEs”)

AND

(“junior high school students” OR “senior high school students” OR adolescent OR teenager). The details of the search terms used are listed in **Table 1**, reflecting the rigor and accuracy of our research method. This study was independently completed by two investigators (ANW and YYP), and if there were any disagreements, the third researcher (TW) would be consulted. The data underwent supervision and final review by TW, while the analysis was carried out by ZBC, QBZ, and ANW.

### 2.2 Eligibility criteria

The inclusion criteria for relevant studies were defined using the PICOS framework. The Population (P) comprised adolescents and children aged 5 to 18 years diagnosed with NDDs (e.g., ASD, ADHD). Those with comorbidities, such as AuDHD, were not included in this study. The Intervention (I) involved various types of PE administered to the experimental group. The Comparison (C) conditions included no-exercise or no treatment controls (NT), standard care or routine activities (RT), and activity/attention placebo controls (AP). The Outcomes (O) primarily assessed symptoms of depression and anxiety in adolescents and children with NDDs. The *Study Design (S)* included randomized controlled trials (RCTs) that provided adequate statistical data (e.g., means, sample sizes, and standard deviations [SDs]) for inclusion in the meta-analysis. Studies without a control group or those lacking between-group comparisons (such as single-case reports or pre-experimental designs) were excluded from the meta-analysis.

Exclusion criteria were as follows: non-English publications, grey literature (e.g., dissertations, unpublished papers, and reviews), studies involving adult or animal subjects, studies with unextractable data, repeated publications, or inaccessible full texts.

### 2.3 Data extraction

Reference management and screening were carried out using EndNote 20, while two researchers (ANW and YYP) independently extracted detailed data using standardized Excel-based forms. In instances of disagreement, a third researcher (TW) was consulted to resolve discrepancies. Data analysis was conducted by QBZ, ANW, and YYP, under the supervision and review of TW and ZBC. Key variables, including SDs, means, and sample sizes, from both intervention and control groups were input into Review Manager 5.3 (32).

Given the expected heterogeneity among the included articles, due to the variety of PE interventions, a random-effects model was utilized for meta-analysis pooling. To standardize results across different depression and anxiety scales, effect sizes were calculated using the standardized mean difference (SMD), specifically Hedges’ g, with correction for small sample bias and presented alongside 95% confidence intervals (CI) (33). Heterogeneity was judged using the I² statistic (32). When significant heterogeneity was detected (I² > 50%), subgroup or sensitivity analyses were conducted to explore potential sources of variation (34).

### 2.4 Methodological quality assessment

The methodological rigor of the selected studies was determined utilizing the “Physiotherapy Evidence Database” (PEDro) scale (35), a validated and commonly adopted instrument for evaluating the quality of RCTs and non-randomized studies (NRS). The PEDro scale has previously been shown to be effective in appraising PE intervention studies involving adolescents and children with NDDs (19, 36). The scale comprises 11 items addressing eligibility, randomization, allocation concealment, and blinding procedures (37), with overall scores from 0 to 10.

It is noteworthy that, owing to the nature of PE interventions, achieving blinding of participants and therapists is often impractical. As highlighted in earlier reviews, this limitation can complicate the scoring of certain PEDro items, particularly those related to blinding (38). Considering these constraints, we adopted a three-tier classification system, consistent with previous assessments: studies scoring 6 or higher were categorized as high quality, reflecting strong methodological rigor; scores of 4–5 were deemed moderate quality, indicating generally sound but not comprehensive design; and scores of 3 or below were considered low quality, suggesting substantial methodological limitations. This approach facilitates a more nuanced understanding of study quality and informs the interpretation of intervention outcomes.

## 3 Results

### 3.1 Study selection

**Figure 1:**
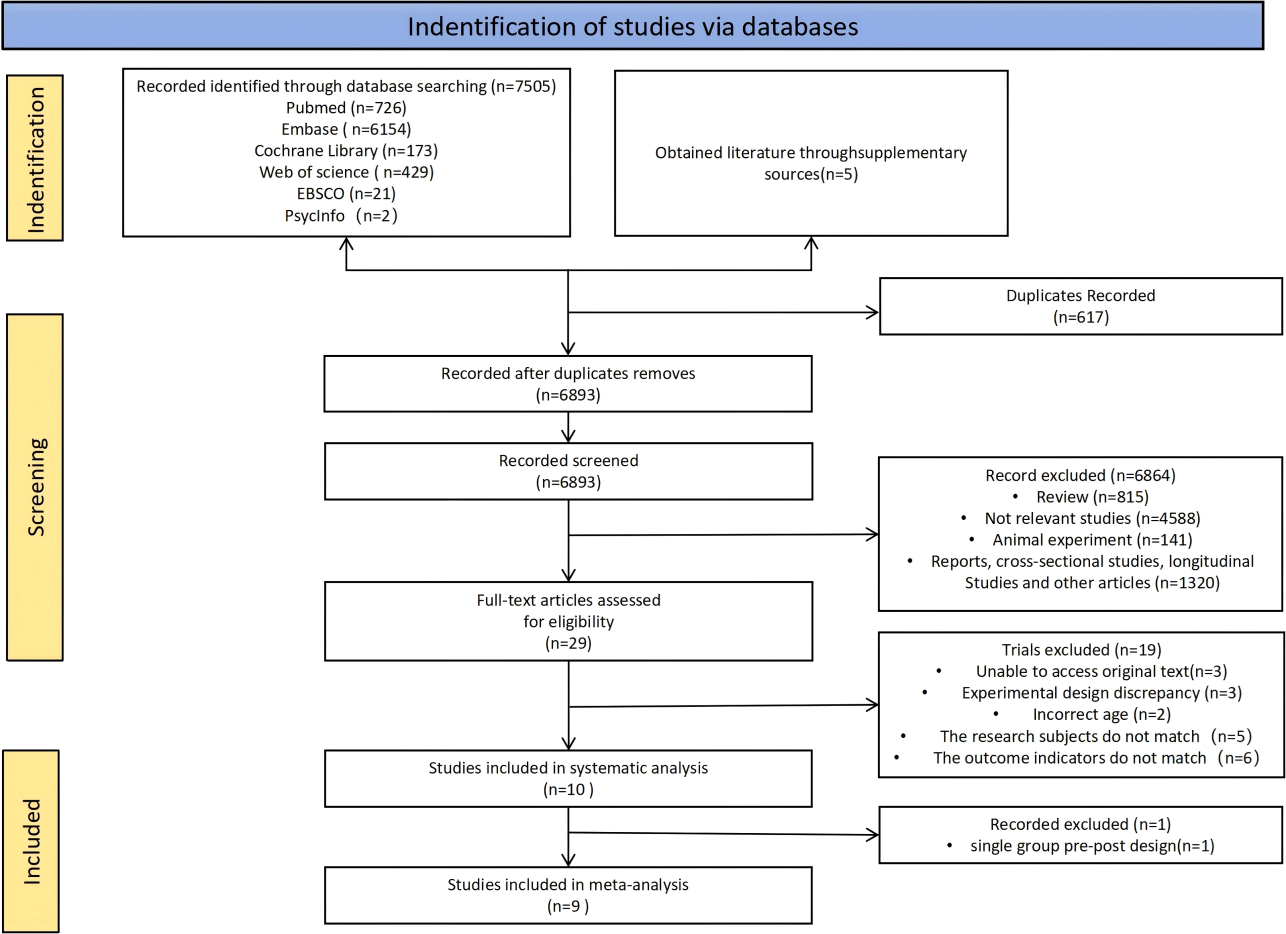
Flow chart of literature retrieval.

**Figure 1** shows the process of search and selection for the studies included in this review. A total of 7,505 articles were initially identified across the databases: 726 from PubMed, 6,154 from Embase, 173 from Cochrane, 429 from WoS, 21 from EBSCO, and 2 from PsycINFO. Additionally, 5 studies were obtained through manual searches or supplementary sources. After removing duplicate records, 6,893 unique articles remained. Among them, 6,864 were excluded during the title and abstract screening for not meeting the inclusion criteria.

The full texts of the remaining 29 studies were assessed, leading to the exclusion of 19 studies for the following reasons: (1) full text unavailable (n = 3); (2) inappropriate study design (n = 3); (3) participant age outside the specified range (n = 2); (4) non-matching study population (n = 5); and (5) outcome measures not aligned with the research focus (n = 6). Ultimately, 10 studies were included in the systematic review. After excluding one single-group pre-post study, 9 studies met the criteria for inclusion in the meta-analysis.

### 3.2 Study characteristics

**Table 2** outlines the key characteristics of the included articles. Among them, one study employed a single-group pre-post design (39), while the remaining nine utilized RCT designs (40–48). The control conditions in the RCTs varied: three studies used a no-intervention control group (43, 45, 48), four applied regular treatment or routine PE (41, 44, 46, 47), and two included an attention or placebo control group (40, 42). Participants across all studies ranged in age from 5 to 18 years.

**Table 2:** Descriptive characteristics of included studies.

Included studies	Country	Sample size (IG/CG)	Diagnosis(severity)	Diagnostic criteria	Gender	Medication	Socioeconomic background	Age	Intervention	Duration (weeks)	Times (min)	Frequency (times /week)	Outcome	Control arm
Carey, 2022 (39)	Ireland	20	ASD (moderate to severe)	GARS	Male	-	-	5-18	Movement skills (Mixed)	16	60	3	ASC-ASD	-
Dhingra, 2025* (40)	India	38(19/19)	ASD (mild to moderate)	-	Male and female	-	-	7-13	Aerobic Training (Mixed)	8	30	3	SCARED	AP
García,2016* (41)	Spain	14(9/5)	ADHD (-)	DSM-IV-TR	Male and female	-	Ordinary schools in urban settings	7-14	Equestrian therapy (Monotypic)	12	45	2	BASC	RT
Gehricke, 2022* (42)	USA	148(76/72)	ASD (-)	the Autism Treatment Network	Male	-	Latino and rural families	6-12	Exercise (Mixed)	8	40-50	3	CBCL DSM-5 anxiety subscale	AP
Hattabi,2022* (43)	Tunisia	40(20/20)	ADHD (-)	K-SADS-PL	Male and female	-	-	9-12	Swimming (Monotypic)	12	90	3	CBCL	NT
Jensen, 2004* (44)	Australia	19(11/8)	ADHD (-)	DSM-IV	Male	Do not take any medication	middle to lower-middle	8-13	Yoga (Monotypic)	20	60	1	CRS	RT
Liu, 2025* (45)	China	80(40/40)	ADHD (-)	reported by their parents	Male and female	Do not take any medication	-	12-17	Aerobic Training (Mixed)	12	60	1	DASS- 21	NT
Pan, 2016* (46)	China	21(16/16)	ADHD (0-2)	DSM-IV	Male	maintain their current pharmacological treatment	-	6-12	Table tennis (Monotypic)	12	60	2	CBCL	RT
Sabzi,2021* (47)	Iran	40(20/20)	ADHD (2-3)	DSM-IV	Male	-	-	9.45 ± 0.5	Water Treadmill (Monotypic)	8	30	3	CPRS-R	RT
Silva,2019* (48)	Brazil,	20(10/10)	ADHD (-)	DSM-IV	Male and female	-	-	11-14	Swimming (Monotypic)	8	45	2	CDI/BAI	NT

AP, activity/attention placebo; ASC-ASD, the Anxiety Scale for Children with Autism Spectrum Disorder; BAI, the Beck inventory; BASC, the Behavioral Assessment System for Children; CBCL, Child Behavior Checklist; CDI, Child Depression Inventory; CG, control group; CPRS-R, Conners’ Parent Rating Scale-Revised; CRS, Conners Rating Scales; DASS- 21, Chinese version of the Depression Anxiety Stress Scale- 21; DSM-IV, diagnostic and Statistical Manual of Mental Disorders; GARS, Gilliam Autism Rating scale - second Edition; IG,International group; K-SADS-PL,the Kiddie-Schedule of Affective Disorders and Schizophrenia Present and Lifetime Version; NT, no-treatment control; RT=Routine treatment; SCARED,The SCARED-P and SCARED-C scales.

* Study included in the meta-analysis.

Regarding diagnoses, three studies focused on participants with ASD (39, 40, 42), while seven targeted individuals with ADHD (41, 43–48). From the diagnostic criteria for children and adolescents with neurodevelopmental disorders, five studies used DSM-IV (Diagnostic and Statistical Manual of Mental Disorders) for diagnosis, one study used GARS (Gilliam Autism Rating Scale - Second Edition) for diagnosis, one study used the Autism Treatment Network for diagnosis, one study used K-SADS-PL (Kiddie-Schedule of Affective Disorders and Schizophrenia Present and Lifetime Version) for diagnosis, and one study was diagnosed by their parents. The severity of the participants’ neurodevelopmental disorders varied, and most of the studies did not report their socio-economic background, comorbid conditions, and medication use. The details are shown in **Table 2**. The intervention types were categorized as either single-mode interventions (e.g., swimming or equestrian therapy) (41, 43, 44, 46–48) or mixed-mode programs involving multiple movement-based activities (39, 40, 42, 45). Intervention durations ranged from 8 to 20 weeks, with session frequencies of 1 to 3 times per week, and individual sessions from 30 to 90 minutes.

### 3.3 Quality assessment

**Table 3** provides a detailed assessment of the methodological quality of the articles included in this review. All studies met a minimum of three core criteria, reflecting a baseline level of methodological soundness. On the whole, the studies demonstrated acceptable quality, with an average PEDro score of 6.56, suggesting a strong methodological framework. Participant eligibility criteria were clearly stated across all studies, supporting appropriate sample selection aligned with the research aims. Additionally, high retention rates throughout the intervention phases helped ensure data reliability and reduced the risk of bias due to participant dropout. However, we have noticed that in most of the studies, the procedure of conducting blind tests for both the participants and the evaluators was rarely carried out. Because if blinding procedures are not followed, the intervention cannot be carried out.

**Table 3:** Methodological quality assessment for included studies.

Included studies	Eligibility criteria	Random allocation	Allocation concealment	Similar at baseline	Subject blinded	Therapist blinded	Assessor blinded	Dropout rate	Intention-to-treat analysis	Between-group comparison	Points measures	Total score	Overall study quality
Carey, 2022 (39)	1	0	0	1	0	0	0	1	1	1	1	5	Adequate
Dhingra, 2025* (40)	1	1	1	1	0	1	0	1	0	1	1	7	High
García,2016* (41)	1	1	0	1	0	0	0	1	1	1	1	6	High
Gehricke, 2022* (42)	1	1	0	1	0	0	0	1	1	1	1	6	High
Hattabi,2022* (43)	1	1	1	1	0	0	0	1	1	1	1	7	High
Jensen, 2004* (44)	1	1	0	1	0	0	0	1	1	1	1	6	High
Liu, 2025* (45)	1	1	1	1	0	0	0	1	1	1	1	7	High
Pan, 2016* (46)	1	1	0	1	0	0	0	1	1	1	1	6	High
Sabzi,2021* (47)	1	1	1	1	0	0	0	1	1	1	1	7	High
Silva,2019* (48)	1	1	1	1	0	0	0	1	1	1	1	7	High

Yes = 1; No = 0. ≤3 are considered ‘poor’, 4–5 are considered ‘adequate’, ≥6 are high.

* Study included in the meta-analysis.

### 3.4 Meta-analysis

To assess the effect of PE on NEs in adolescents and children with NDDs, nine RCTs were included using anxiety and/or depression as outcome measures. An initial heterogeneity analysis revealed considerable variability across studies (I² = 79%, p < 0.01), indicating substantial differences in study characteristics. This high level of heterogeneity could be attributed to variations in participant demographics, intervention types, research settings, and statistical methodologies, as well as potential publication bias. These factors suggest that the pooled effect size may not fully capture the true intervention impact, thus restraining the findings’ generalizability.

**Figure 2:**
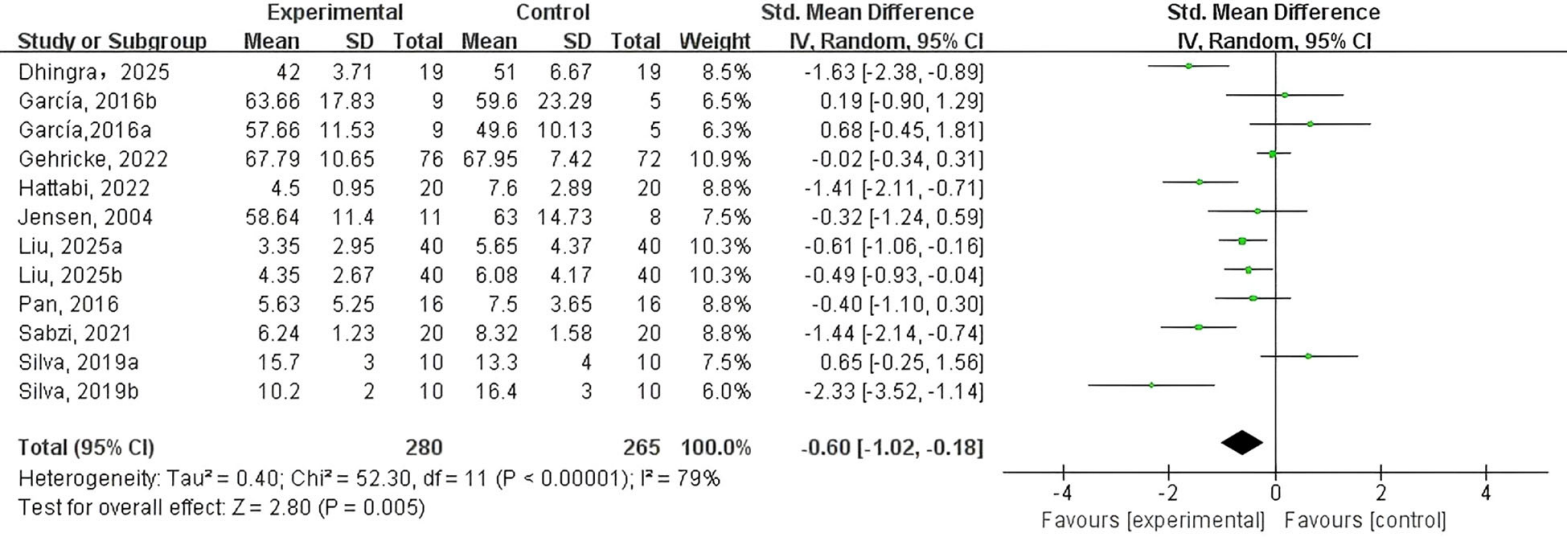
Forest plot of the effect of PE on negative emotions.

To address this, a random-effects model was employed, and subgroup analyses were conducted to reduce heterogeneity. As illustrated in **Figure 2**, the meta-analysis demonstrated that the PE intervention group exhibited a suggestively greater drop in NEs relative to the control group (SMD = -0.60, 95% CI: -1.02 to -0.18, p < 0.01, Z = 2.80, I² = 79%). These findings indicate that PE interventions can meaningfully lessen symptoms of depression and anxiety in adolescents and children with NDDs.

### 3.5 Publication bias

**Figure 3:**
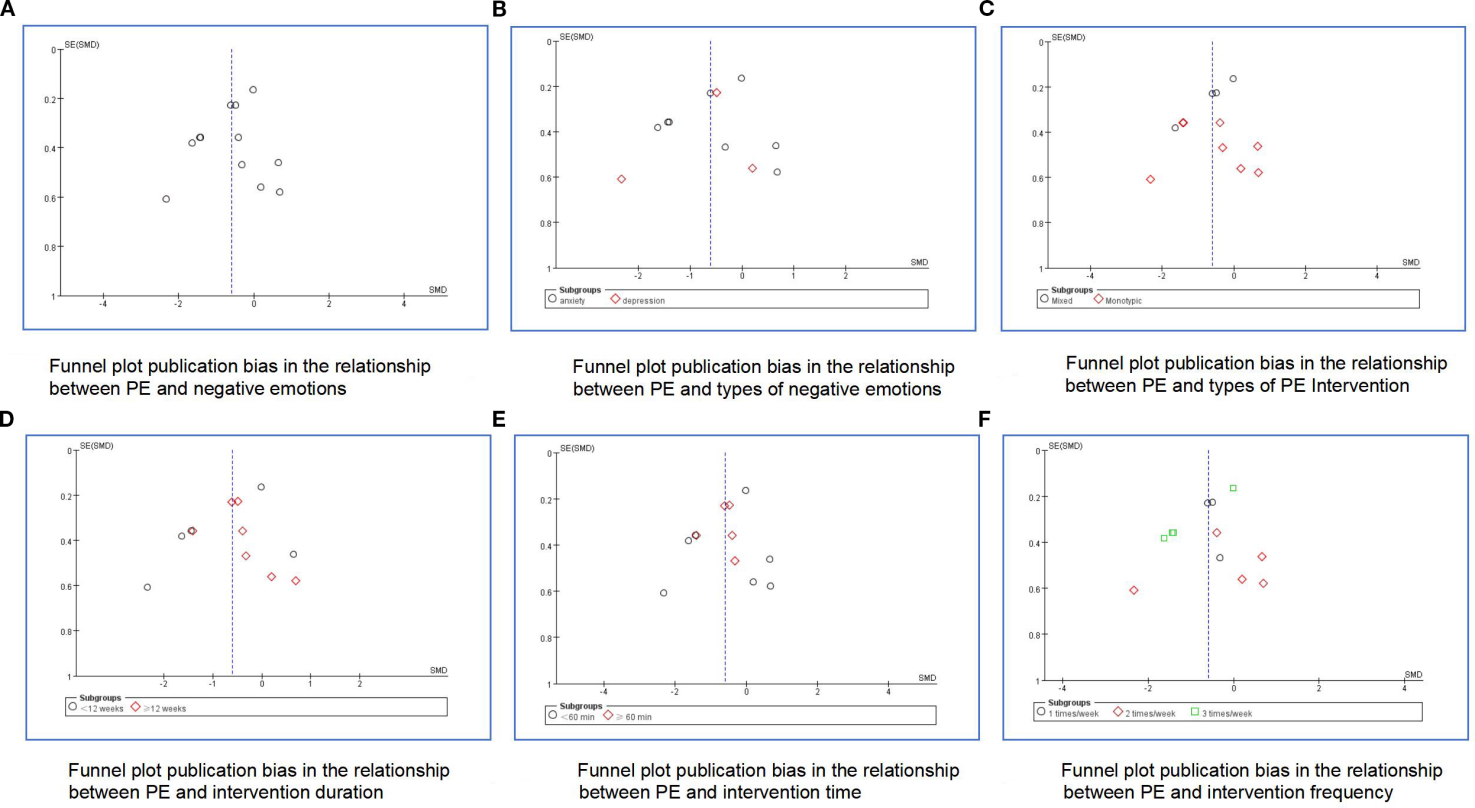
Funnel plots of publication bias for the meta-analysis. **(A)** overall negative emotions, **(B)** types of negative emotions, **(C)** types of PE intervention, **(D)** intervention duration, **(E)** intervention time, **(F)** intervention frequency.

Publication bias was estimated via a funnel plot’s visual inspection (**Figure 3**). The plot displayed noticeable asymmetry, suggesting the presence of potential publication bias. This imbalance implies that smaller studies reporting non-significant outcomes may be understated, which may result in an inflated estimation of the overall effect size in the meta-analysis. In addition to publication bias, the asymmetry could also be influenced by methodological inconsistencies across studies, such as differences in sample size, study design, and participant characteristics. The variability in effect sizes, particularly among smaller trials, may further contribute to the distortion observed in the funnel plot.

### 3.6 Sensitivity analysis

To further investigate potential heterogeneity’s sources, sensitivity analyses were performed by sequentially removing each study from the meta-analysis. This approach allowed for the evaluation of individual studies’ influence on the overall findings. Key factors, including study design, sample size, and methodological quality, were taken into account during this process. The results remained consistent across all iterations, implying that no single study disproportionately impacted the overall outcome. This stability reinforces the reliability and robustness of the pooled effect size.

### 3.7 Subgroup analysis

To investigate factors that have an influence on the effects of PE on NEs, a subgroup analysis was conducted (**Table 4**). The analysis aimed to scrutinize the moderating roles of several variables, including the type of negative emotion (depression or anxiety), intervention type (single-mode or mixed), intervention duration (<12 weeks or ≥12 weeks), session length (<60 minutes or ≥60 minutes), and intervention frequency (once, twice, or three times per week).

**Table 4:** Subgroup analyses based on the primary meta-analysis.

Subgroup analysis	K	SMD	95%CI	*p* value	Heterogeneity	Test for subgroup difference
Primary meta-analysis	9	-0.60	-1.02 to -0.18	p=0.005<0.01	Chi^2^ = 52.30, df = 11 (p<0.00001), Z = 2.80, I^2^ = 79%	
Type of Negative Emotions						
Depression	3	-0.82	-2.00 to -0.36	*p = 0.17*	Chi^2^ = 445.61, df = 2 (p= 0.006), Z = 1.37, I^2^ = 81%	Chi^2^ = 0.16, df = 1 (P = 0.69), Z = 2.64, I^2^ = 0%
Anxiety	8	-0.56	-1.11 to -0.00	*P = 0.05*	Chi^2^ = 42.53, df = 7 (p<0.00001), Z = 1.97, I^2^ = 83%	
Type of PE Intervention						
Monotypic	6	-0.56	-1.24 to 0.12	*p = 0.11*	Chi^2^ = 33.49, df = 7 (p<0.00001), Z = 1.62, I^2^ = 79%	Chi^2^ = 0.01,df = 1 (P = 0.91), Z = 2.80, I^2^ = 0%
Mixed	3	-0.61	-1.16 to -0.07	P = 0.03<0.05	Chi^2^ = 16.94, df = 3 (P = 0.0007), Z = 2.20, I^2^ = 82%	
Duration						
<12 weeks	4	-0.91	-1.88 to 0.06	P = 0.07	Chi^2^ = 39.47, df = 4 (P<0.00001), Z = 1.84, I^2^ = 90%	Chi^2^ = 0.69, df = 1 (P = 0.41), Z = 2.80, I^2^ = 0%
≥12 weeks	5	-0.47	-0.85 to -0.08	P = 0.02<0.05	Chi^2^ = 12.63, df = 6 (P = 0.05), Z = 2.37, I2 = 5^2^%	
Time						
<60min	5	-0.56	-1.34 to 0.23	P = 0.016	Chi^2^ = 44.13, df = 6 (p<0.00001),Z = 1.39, I^2^ = 86%	Chi^2^ = 0.04,df=1(P = 0.84), Z = 2.80, I^2^ = 0%
≥60min	4	-0.64	-0.97 to -0.31	P = 0.0001<0.001	Chi^2^ = 6.02, df = 4 (p = 0.20), Z = 3.83, I^2^ = 79%	
Frequency						
1 times/week	3	-0.53	-0.82 to -0.23	P = 0.0006<0.001	Chi^2^ = 0.35, df = 2 (p=0.84),Z = 3.45, I^2^ = 0%	Chi^2^ = 1.77, df = 2 (P = 0.41), Z = 2.80, I^2^ = 0%
2 times/week	5	-0.22	-1.17 to 0.73	P = 0.65	Chi^2^ = 18.80, df = 4 (p = 0.00009), Z = 0.45, I^2^ = 90%	
3 times/week	4	-1.09	-2.03 to -0.15	P = 0.02<0.05	Chi^2^ = 30.55, df = 3 (p<0.00001), Z = 2.27, I^2^ = 90%	

K, Number of trials; SMD, Standardized Mean Difference; CI, Confidence Interval; PE, Physical Exercise.

**Figure 4:**
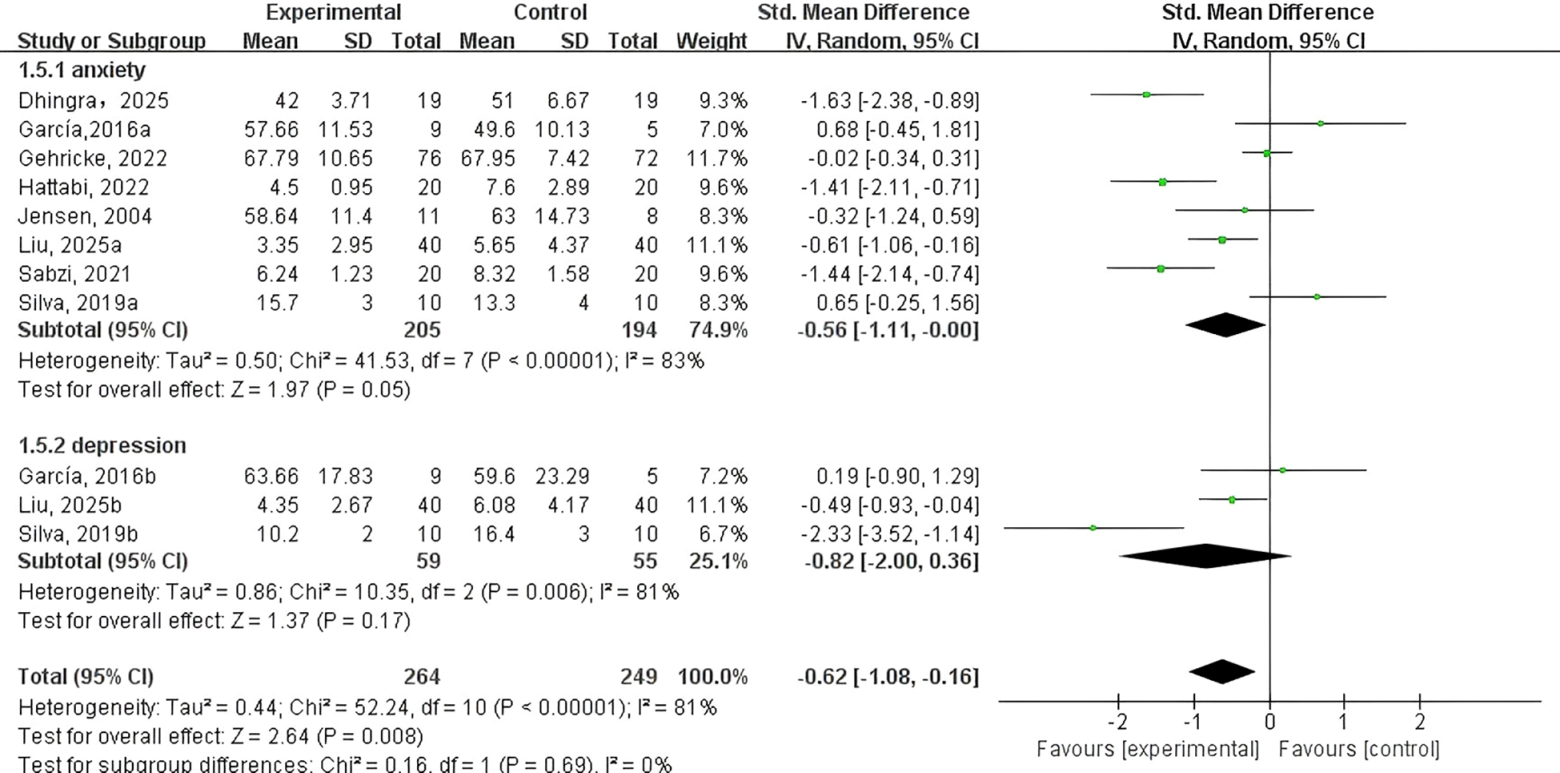
Forest plot of a meta-analysis of the relationship between PE and types of negative emotions.

Types of NEs (**Figure 4**). Three studies (41, 45, 48) reported data on the effects of PE interventions on depression, while 8 studies (40–45, 47, 48) examined effects on anxiety in adolescents and children with NDDs. A random-effects model was utilized for the meta-analysis. The effect was most evident for anxiety (SMD = -0.56, 95% CI: -1.11 to -0.00, p = 0.05, I² = 83%), suggesting a potential reduction in anxiety symptoms that approaches statistical significance. Conversely, the effect on depression was not statistically significant (SMD = –0.82; 95% CI: –2.00 to –0.36; p = 0.17; I² = 81%).

**Figure 5:**
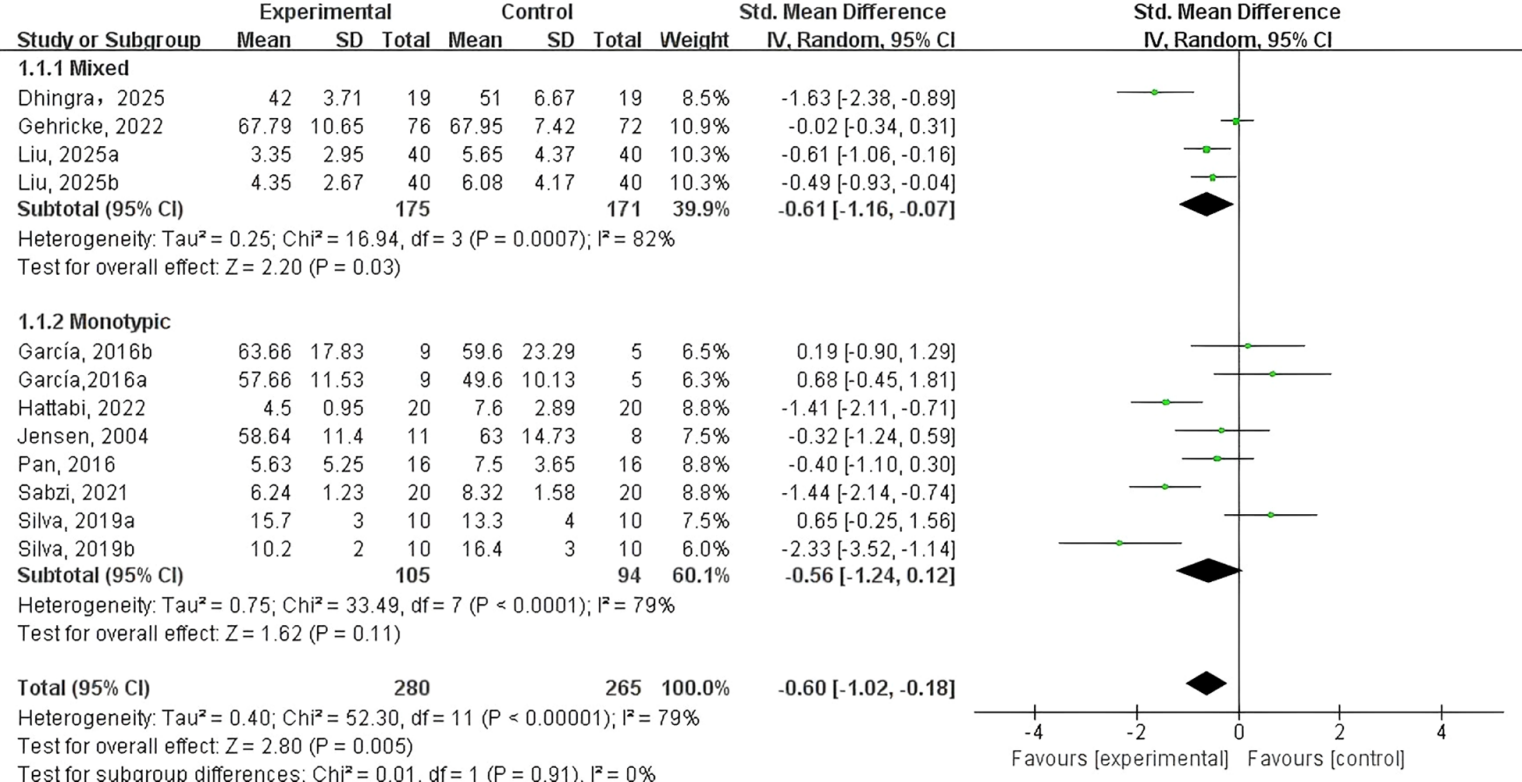
Forest plot of a meta-analysis of the relationship between type of PE intervention and negative emotions.

Types of PE intervention (**Figure 5**). Data on monotypic exercise were reported in eight studies, while four studies examined mixed exercise. Subgroup analysis revealed that mixed exercise significantly alleviated NEs (SMD = -0.61, 95% CI: -1.16 to -0.07, p < 0.05, I² = 82%). In contrast, monotypic exercise showed no statistically significant effect on NEs among adolescents and children with NDDs (SMD = -0.56, 95% CI: -1.24 to 0.12, p = 0.11, I² = 79%).

**Figure 6:**
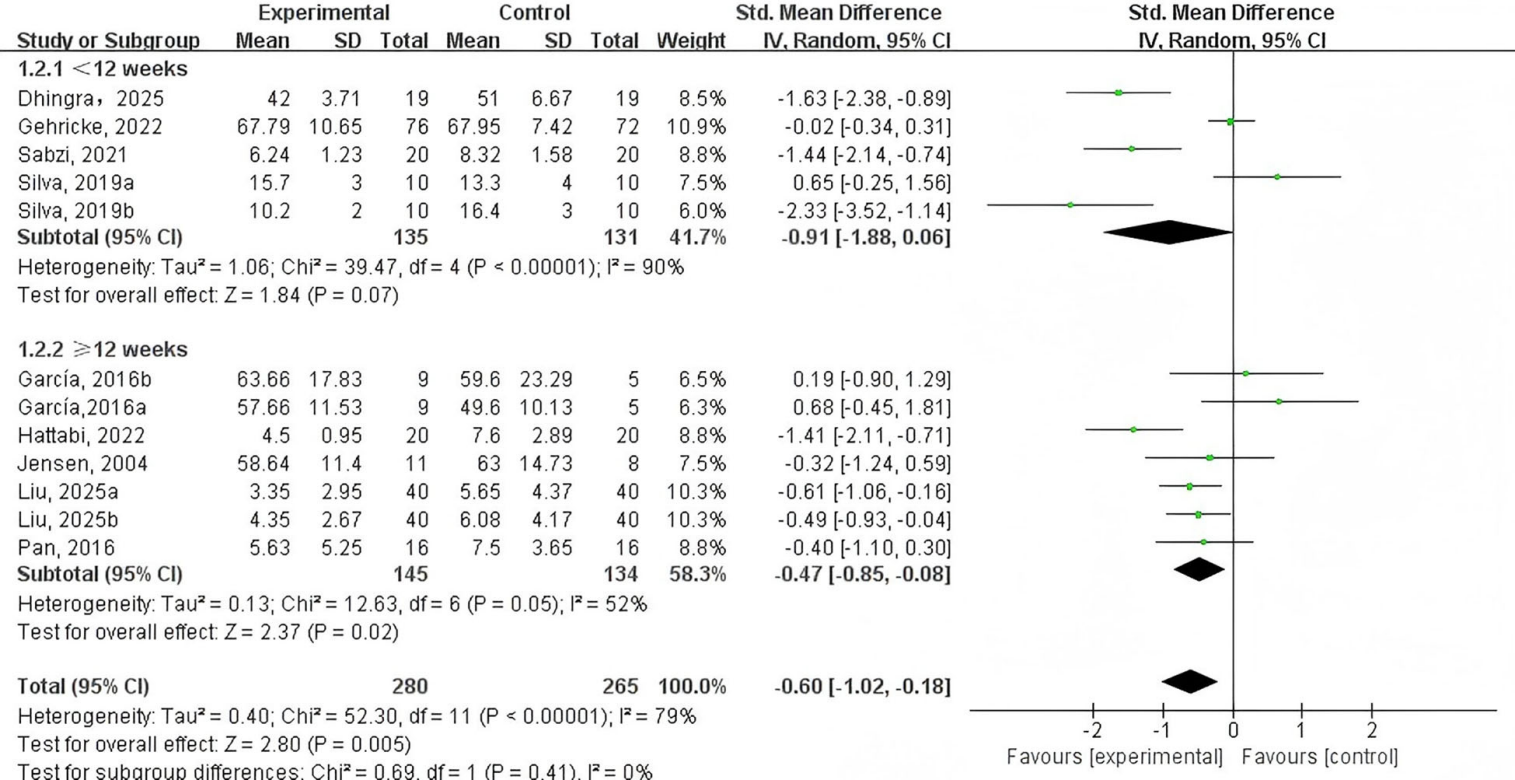
Forest plot of a meta-analysis of the relationship between PE intervention duration and negative emotions.

PE intervention duration (**Figure 6**). Four studies (40, 42, 47, 48) examined PE interventions lasting less than 12 weeks, while 5 studies (41, 43–46) focused on interventions of 12 weeks or longer. Findings showed that PE interventions lasting 12 or >12 weeks significantly reduced NEs in adolescents and children with NDDs (SMD = -0.47, 95% CI: -0.85 to -0.08, p = 0.02, I² = 52%). In contrast, shorter interventions of under 12 weeks (SMD = -0.91, 95% CI: -1.88 to 0.06, p = 0.07, I² = 90%) did not produce a statistically significant effect.

**Figure 7:**
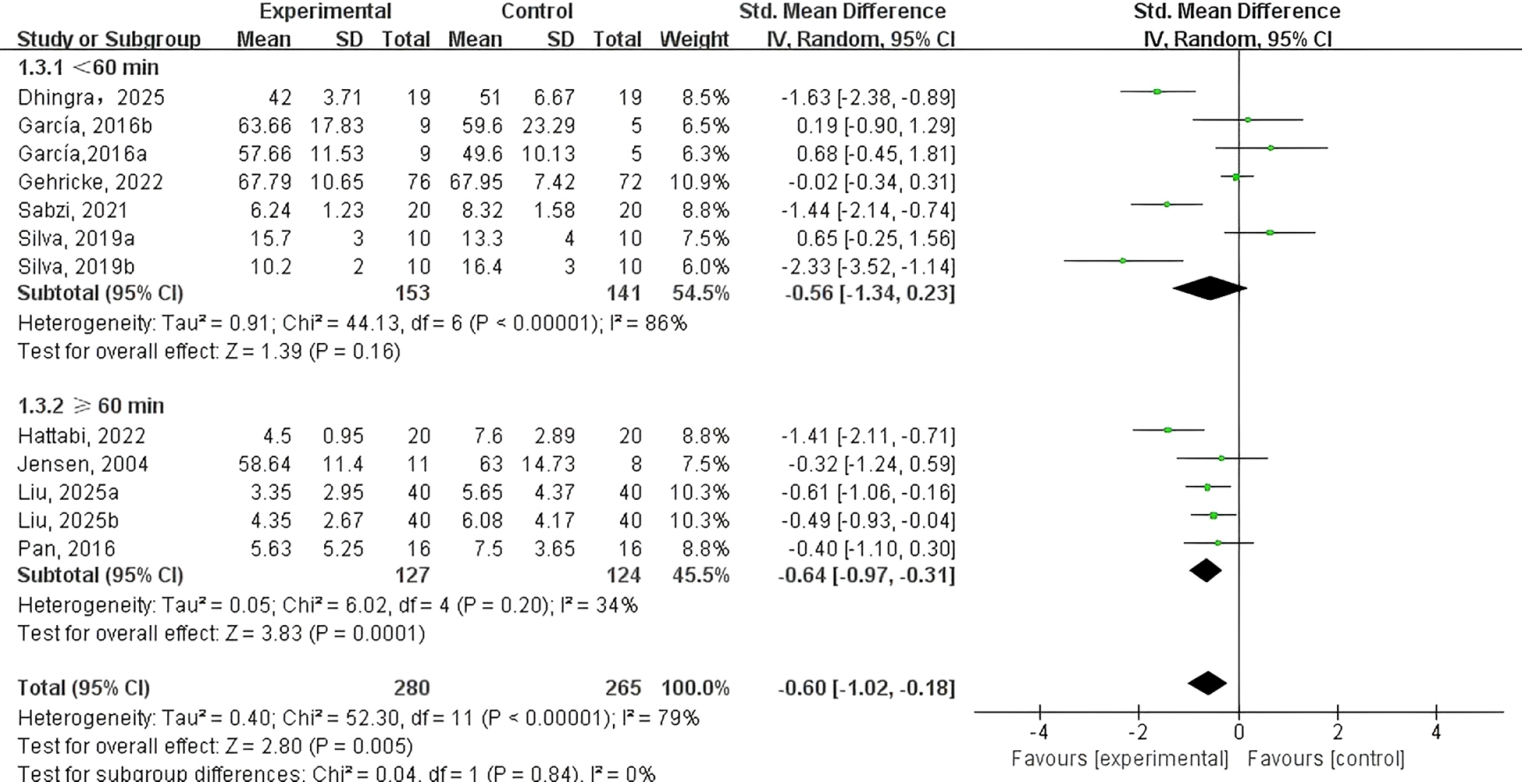
Forest plot of a meta-analysis of the relationship between PE intervention time and negative emotions.

PE intervention time (**Figure 7**). Five studies (40–42, 47, 48) assessed PE interventions lasting under 60 minutes, whereas four studies (43–46) focused on sessions of 60 minutes or more. The analysis showed that interventions shorter than 60 minutes (SMD = -0.56, 95% CI: 1.34 to 0.23, p = 0.016, I² = 86%) did not yield a significant reduction in NEs among adolescents and children with NDDs. In contrast, interventions lasting 60 minutes or longer (SMD = -0.64, 95% CI: -0.97 to -0.31, p < 0.001, I² = 79%) demonstrated a significant positive effect.

**Figure 8:**
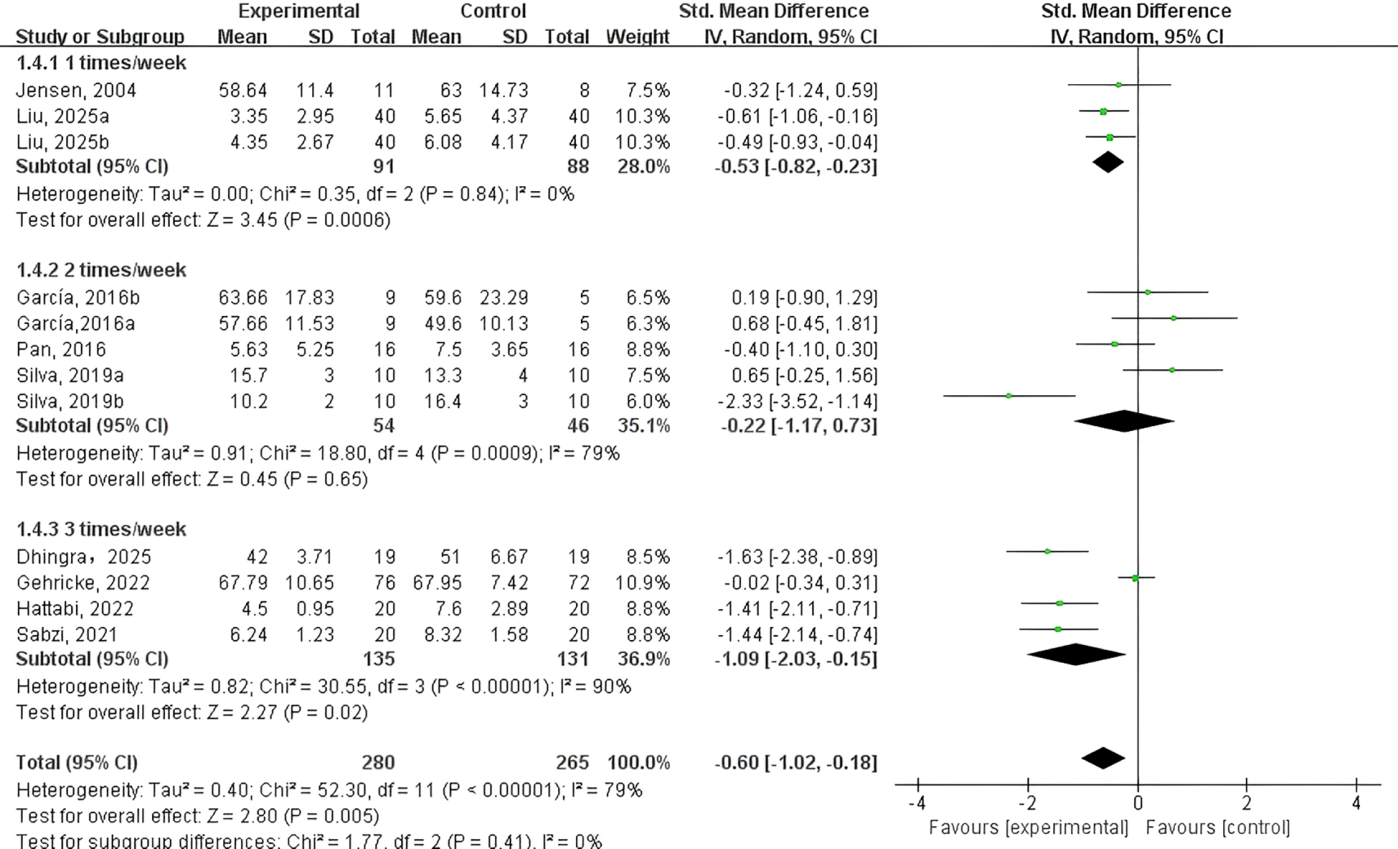
Forest plot of a meta-analysis of the relationship between PE intervention frequency and negative emotion.

PE intervention frequency (**Figure 8**). Two studies (44, 45) examined PE interventions implemented once per week; three studies (41, 46, 48) focused on twice-weekly sessions; and 4 studies (40, 42, 43, 47) reported on interventions performed 3 times per week. The findings suggested that both once-weekly (SMD = -0.53, 95% CI: -0.82 to -0.23, p < 0.001, I² = 0%) and three-times-weekly PE interventions (SMD = -0.22, 95% CI: -1.17 to 0.73, p = 0.65, I² = 90%) showed potential in reducing NEs among adolescents and children with NDDs. However, interventions conducted twice weekly (SMD = -1.09, 95% CI: -2.03 to -0.158, p = 0.02, I² = 90%) did not demonstrate a significant effect. Overall, once-weekly PE sessions appeared to be the most effective in improving emotional well-being in this population.

## 4 Discussion

This research investigates the effects of PE on NEs in adolescents and children with NDDs. Out of an initial pool of 7,510 records, nine studies were ultimately included in the meta-analysis. As all selected studies were RCTs involving PE interventions, full blinding was not feasible. Consequently, the absence of blinding was not considered a marker of low study quality, as such an assessment would have been inappropriate. The results demonstrated a significant reduction in NEs among participants engaged in PE. Subgroup analyses further revealed that interventions conducted once weekly, lasting over 60 minutes per session, and maintained for a minimum of 12 weeks were particularly effective in alleviating NEs in this population.

### 4.1 The impact of PE intervention on various types of NEs in adolescents and children with NDDs

The findings of our meta-analysis suggest that PE may be associated with reductions in anxiety in adolescents, though the evidence should be interpreted cautiously given the limited number of studies (n=9) and their heavy focus on ADHD populations (7 of 9 studies). Moreover, several studies have highlighted the anxiolytic benefits of PE in ASD children. For instance, Hillier et al. (49) conducted an eight-week, lower-intensity PE program, delivered once weekly to eighteen adolescents and young adults diagnosed with higher-functioning ASD, which included aerobic, flexibility, balance, and strength exercises. Participants reported notable reductions in both anxiety and cortisol levels during individual sessions; however, these effects did not persist over the entire intervention period. Golsefidi and Hashemi (50) recently administered a four-week motor skills program, held 3 times per week for ten ASD children aged 6–10, and found significant reductions in their self-reported anxiety. These findings support the potential of PE as a non-invasive and cost-effective intervention for anxiety in ASD children. Our meta-analysis reinforces this conclusion, confirming the efficiency of PE in decreasing anxiety among adolescents.

In contrast, our meta-analysis produced inconclusive findings regarding the effect of PE on depressive symptoms. This aligns with the conclusions of a previous meta-analysis (51) but should be interpreted with caution, as other studies (20) have reported potential antidepressant effects of PE may significantly reduce depression. The variability in outcomes concerning depressive symptoms could be ascribed to variations in study design and participant characteristics, or intervention protocols. First, variations in exercise modalities, duration, intensity, and frequency across different studies could result in heterogeneity, contributing to contradictory outcomes. Second, differences in measurement tools and criteria for depressive symptoms could lead to inconsistent sensitivity or specificity in detecting intervention effects. Additionally, depression in NDDs may involve complex and multifaceted mechanisms influenced by individual neurobiological and psychosocial factors, meaning exercise might not uniformly impact all adolescents. Lastly, the severity and type of NDDs, as well as comorbidities, could modulate the therapeutic potential of exercise, resulting in varied effectiveness across distinct participant populations. Therefore, the disparity between findings highlights the requirement for further standardized investigation to clarify the contexts and conditions under which PE most effectively alleviates depressive symptoms in this unique demographic.

### 4.2 The effects of types of PE intervention on the NEs of adolescents and children with NDDs

This study investigates whether different types of PE interventions influence the effectiveness of reducing NEs in adolescents and children with NDDs. Our findings suggest that mixed-type exercise interventions are more effective than monotypic (single-form) exercises in alleviating NEs, which aligns with previous research (52). The mixed exercises included in the analyzed studies comprised a combination of movement skill training, aerobic activities, and physical conditioning. For example, Carey’s study focused on developing fundamental motor skills such as striking, dodging, kicking, balancing, landing, jumping, catching, and throwing with hands or equipment. These complex movements were broken down into manageable components and practiced under the supervision of instructors or special education aides, such as learning to throw and catch balls (39). In Dhingra’s study, the intervention incorporated activities like walking, jumping, static cycling, running, and lateral movement drills, followed by a five-minute cool-down session involving shoulder and lower back stretches, child’s pose, and trampoline exercises (40). In contrast, monotypic interventions involved only one type of PE, such as swimming (43, 48), yoga (44), or table tennis (46). For ADHD children, enhancing enjoyment in physical activities plays a crucial role in fostering self-efficacy. The significantly greater improvement in NEs observed with mixed exercises may be due to their flexible and engaging structure, which provides children with ADHD more consistent opportunities for positive emotional reinforcement during participation (52).

### 4.3 PE intervention duration’s impact on NEs of adolescents and children with NDDs

This study examined whether the duration of PE interventions influenced their effectiveness in alleviating NEs among adolescents. The included articles were divided into two subgroups based on intervention length: less than 12 weeks and 12 weeks or longer. Analysis of subgroups revealed that interventions extending for 12 weeks or longer demonstrated markedly greater effectiveness compared to those of shorter duration, consistent with findings from prior research (52). Evidence suggests that medium- to long-term exercise programs tend to produce better outcomes; however, further validation through high-quality, large-sample RCTs is desired to determine whether the benefits continue or increase with extended durations (52). For instance, Carey’s study demonstrated that a 16-week PE intervention significantly reduced anxiety symptoms in children with Social Anxiety Disorder (SAD) in school settings. Anxiety levels showed a more notable reduction at the 16th week versus the 8th week. Teachers also reported marked improvements in ASC-ASD total scores and subscales related to performance anxiety, arousal, and uncertainty after the 16-week program (39). Similarly, Liu’s study found that aerobic-based PE significantly alleviated anxiety, stress, and depression symptoms, with these benefits persisting for up to three months post-intervention (45). Several factors may account for these sustained effects. One explanation is the “floor effect,” where individuals with initially low baseline performance have more potential for improvement (53). A prior meta-analysis indicated that individuals with clinical diagnoses tend to gain more emotional advantages from PE interventions compared to their typically developing counterparts (54). Given that adolescents and children with NDDs often experience emotional and psychological challenges, exercise-based programs may contribute to improvements in emotional well-being. These improvements could be partially mediated by physiological mechanisms, such as modulation of the sympathetic nervous system and hormonal regulation, that are sometimes observed following aerobic exercise (55). These variations are supposed to be strongly associated with anxiety-related symptoms and other mental health issues, offering a potential explanation for the lasting effects of PE (55).

### 4.4 PE intervention time’s impact on NEs of adolescents and children with NDDs

Our meta-analysis explored the impact of intervention duration on the relationship between PE and NEs in adolescents and children with NDDs. The included studies were categorized into two groups: those with sessions < 60 minutes versus those > 60 minutes. Subgroup analysis revealed that interventions lasting 60 or > 60 minutes notably improved negative emotional outcomes, whereas shorter sessions did not yield a statistically significant effect, findings that are consistent with previous studies (43, 44). These results align with existing public health recommendations, including those from the “American Academy of Pediatrics” and the Canadian government, which advocate for a minimum of 60 minutes of moderate-to-vigorous PE per day to support the mental and physical well-being of adolescents and children. This underscores the essential role of regular, adequately timed PE in enhancing overall health (56). Similarly, the WHO also advises that adolescents and children engage in an average of 60 minutes of moderate to vigorous PE daily to optimize physical, psychological, and cognitive development (57).

### 4.5 PE intervention frequency’s impact on NEs of adolescents and children with neurodevelopmental disorder

This study explored how the frequency of PE interventions influences their impact on NEs in adolescents and children with NDDs. The included studies were grouped based on intervention frequency: 1, 2, and 3 times in a week. Subgroup analysis indicated that both once-weekly and thrice-weekly PE programs were effective in reducing NEs; however, interventions conducted once per week showed the largest effect size. In contrast, twice-weekly programs did not show statistically significant improvements. Several factors may account for this pattern. First, the limited number of studies in the twice-weekly subgroup may have reduced statistical power, potentially resulting in a type II error. Second, characteristics specific to NDD populations—such as sensory sensitivities, social challenges, and heightened stress responses—could make more frequent sessions difficult to tolerate. For many children with ASD or ADHD, even moderate physical activity can be physically and emotionally demanding, and excessive frequency may lead to overstimulation, sensory overload, or fatigue, thereby diminishing potential benefits. For example, Hillier et al. implemented a low-intensity PE program once weekly for 18 adolescents with high-functioning autism, which resulted in a notable decline in self-reported anxiety levels during the intervention (49). The enhanced effectiveness of once-weekly sessions may reflect an optimal balance between routine exposure and psychological recovery. Less frequent sessions can reduce anxiety and fatigue associated with intensive schedules, providing a more predictable, low-pressure environment conducive to emotional regulation. Weekly programs also allow time for adequate recovery, reduce the likelihood of dropouts, and can foster more consistent engagement. Importantly, carefully structured group settings—matching participants by ability and diagnosis—may further improve outcomes by minimizing social stressors and competitive pressure. Therefore, while our findings tentatively suggest that a once-weekly intervention structure may be more effective for improving emotional outcomes, these results should be interpreted cautiously given the small number of studies and potential subgroup heterogeneity. Future research should investigate the optimal frequency of PE interventions, considering sensory, psychological, and social needs of NDD populations.

## 5 Limitations of the review

This review has several notable limitations. First, the limited number of included studies and sample sizes may limit the capacity to make conclusive judgments regarding the efficacy of PE interventions. Second, some studies relied primarily on self-report questionnaires to assess NEs in adolescents and children with NDDs. This approach is susceptible to recall bias and inconsistent reporting, which may compromise the reliability and accuracy of the data. Third, due to the inclusion of less than ten studies, this review was unable to perform certain subgroup analyses or explore moderating variables such as disorder severity or PE intervention intensity. Fourth, most participants in the included studies were adolescents and children diagnosed with ASD or ADHD. To broaden the scope, future investigation should research the link between PE and NEs in youth with other types of NDDs. Fifth, while this meta-analysis focused on depression and anxiety, negative affect also includes other emotional states such as sadness, stress, distress, and anger. Future studies should consider a more comprehensive range of emotional outcomes. Lastly, most included studies provided details only about the monitoring of intervention groups, with limited information on the monitoring procedures for control groups. Future research should address this gap by reporting on exercise duration and providing detailed monitoring protocols for both intervention and control groups.

## 6 Conclusion

This study aimed to synthesize existing evidence on the association between PE and NEs—specifically anxiety and depressive symptoms—among adolescents and children with NDDs, with particular attention to the current limitations and the inconclusive nature of findings related to depressive symptoms. The findings indicate that PE can effectively reduce negative emotional symptoms in this population. Subgroup analysis further revealed that mixed-type exercises performed once per week, for at least 60 minutes per session, and sustained over a period of 12 weeks or more, yielded the most significant improvements in emotional well-being. Nevertheless, several limitations must be acknowledged. These include a limited sample size, potential publication bias, heterogeneity within subgroup analyses, and the reliance on subjective measurement tools lacking objective indicators. Furthermore, many of the included studies lacked standardized protocols, with inconsistencies in cohort characteristics, intervention duration, exercise modalities, and control group monitoring, which complicates interpretation. The variability in study outcomes also warrants caution when interpreting the results. Future research should aim to explore how intervention effects may differ by gender, age, and exercise intensity. It is also recommended that future studies incorporate localized control variables into intervention designs and compare findings across cultural contexts to better understand the global applicability of PE interventions in addressing NEs among youth with NDDs.

## Data Availability

The original contributions presented in the study are included in the article/supplementary material. Further inquiries can be directed to the corresponding authors.
